# Outcomes of Esophageal Atresia at University Tertiary Hospital of Kigali, Rwanda

**DOI:** 10.21203/rs.3.rs-7678868/v1

**Published:** 2025-10-01

**Authors:** Chioma Moneme, Kimberley Duru, Owen Selden, Shaina Twardus, Jordan Gooding, Jean Pierre Habumufasha, Aimable Kanyamuhunga, Ruyun Jin, Tracy Kelly, Sandra Kabagambe, Edmond Ntaganda

**Affiliations:** University of Virginia; University of Virginia; University of Virginia; University of Virginia; University of Virginia; University of Rwanda, University Teaching Hospital of Kigali; University of Rwanda, University Teaching Hospital of Kigali; University of Virginia; University of Virginia; University of Illinois Urbana-Champaign; University of Rwanda, University Teaching Hospital of Kigali

**Keywords:** Rwanda, tracheoesophageal fistula, esophageal atresia, low- and middle-income countries, access to care, pediatric surgical care

## Abstract

**Purpose:**

Mortality associated with EA/TEF has declined in HICs with advances in multidisciplinary care. However, it remains as high as 80% in LMICs. This study examines the current clinical outcomes of neonates with EA/TEF at a tertiary-level hospital in Rwanda following the implementation of care by fellowship-trained pediatric surgeons.

**Methods:**

A retrospective cohort study of neonates with EA/TEF from January 2015 to December 2023. Patient data were collected from medical logbooks for all patients who received surgical treatment at Centre Hospitalier Universitaire de Kigali, Rwanda. Univariable logistic regression was used to identify factors associated with higher 30-day mortality.

**Results:**

56 patients were included. All infants were born at term and, on average, arrived 7 days after birth (6.98 ±5.18). Type C was the most common anomaly (68%). Mortality data were only available for 82.1% of patients. Of this subset, the 30-day mortality was 52.2%. Increased odds of mortality were associated with the presence of any congenital anomaly (p <0.05) and specifically a cardiac congenital anomaly (p<0.05).

**Conclusion:**

This study provides insights into infants with EA/TEF after implementing specialized surgical care, which has helped reduce mortality compared to other LMICs. Targeted quality improvement initiatives for infants with additional associated congenital anomalies could further improve outcomes.

## Introduction

Esophageal atresia with or without tracheoesophageal fistula (EA/TEF) is the most common congenital anomaly of the esophagus, estimated to affect 1 of every 2,500 to 4,000 live births, and results from the failure of separation of the foregut. (1–4). EA/TEF is known to occur in isolation but often occurs in association with other congenital and chromosomal anomalies including VACTERL association (vertebral, anal, cardiac, tracheoesophageal, renal and limb anomalies), CHARGE association (coloboma, heart anomalies, choanal atresia, retarded growth, genital anomalies, and ear anomalies), cardiac defects, trisomy 18 and trisomy 21 with additional anomalies seen in up to 55% of congenital EA/TEF cases. (2,3,5). It confers an elevated risk of mortality and morbidity during the early neonatal period due to aspiration, respiratory failure, and failure to thrive (6).

Congenital disorders are one of the leading causes of death in children under the age of 5 worldwide. (7) Over 90% of deaths associated with congenital anomalies occur in low -and middle-income countries (LMICs). (8) Evolution in management of EA/TEF has been well described, and early surgical intervention remains the mainstay of management. (9) With advances in pediatric surgery expertise and operative techniques, mortality rates in high-resource settings are as low as 5%. (6)(10) Conversely, management of infants with EA/TEF in low-resource settings continues to present a significant challenge, with mortality rates up to 80%. (11)

Whereas mortality in high-resource settings in infants with EA/TEF is highly associated with additional congenital anomalies, delays in care delivery, sub-optimal health infrastructure, and lack of skilled health care providers significantly affect mortality rates in LMICs(10)(12). Nonetheless, healthcare delivery is not static in low-resource settings, and recruitment of skilled providers, relevant training, and infrastructure improvement may alter outcomes. Few studies exist in the literature that describe outcomes in infants with EA/TEF in LMICs where fellowship-trained pediatric surgeons are available. This study aimed to evaluate the surgical management, anatomic classification distribution, and outcomes of infants with EA/TEF at Centre Hospitalier Universitaire de Kigali (CHUK), a high-volume tertiary center and teaching hospital in Rwanda, with fellowship-trained pediatric surgeons.

## Methods

### IRB ethical approval

This study received Institutional Review Board approval from the CHUK Office of Clinical Education and Research and the University of Virginia (UVA) Health Sciences Research Institutional Review Board (#HSR230210). Patient identifiers were not collected and were only used to identify patient medical files.

### Patient selection

This is a single-center, retrospective cohort study of neonates who presented to CHUK with esophageal atresia, with or without a tracheoesophageal fistula, from 2015 to 2023. Patients were identified from operating theatre logbooks with a documented preoperative diagnosis of EA/TEF. All patients who were evaluated and treated for EA/TEF were included. Patients who had any operative intervention before arrival at CHUK were excluded.

### Clinical and demographic data collection

Data was collected from paper medical records available from the CHUK Records and Archives department by matching patient medical record numbers from operating theatre logbooks with archive codes for patients that met our inclusion criteria. The patienťs full name and date of birth were used as additional identifiers to ensure data was obtained from the correct paper chart. Demographic characteristics collected included age, sex, province, district of primary residence, health insurance status, type of health insurance, and place of birth. Preoperative variables obtained included maternal prenatal care, prenatal diagnosis of an additional congenital anomaly, birth weight, age at arrival to CHUK, weight upon admission, and additional congenital anomalies. Patient-specific perioperative clinical characteristics included the number of days between initial admission and surgical repair, weight at the time of surgical repair, surgical repair approach, EA/TEF Gross classification type, length of stay, and any complications that occurred following surgical repair.

### Outcomes

The primary outcome was 30-day mortality, which was determined from either a filed death certificate, ongoing inpatient status, or recorded outpatient follow-up at CHUK, which was greater than 30 days postop. Secondary outcomes of interest were complications in the postoperative period related to the EA/TEF repair, including anastomotic leaks, fistula recurrence, esophageal strictures, and stenosis. Other complications unrelated to the technical aspects of the repair included sepsis, pneumonia, and respiratory failure.

### Data Analysis

Continuous variables were summarized using the mean with standard deviation (SD) or the median with interquartile range (IQR), and compared between groups using either the t-test or the Wilcoxon Rank Sum test, as appropriate. Categorical variables were presented as counts and proportions, and compared between groups using the Chi-square test or Fisher’s exact test, as applicable. Missing data was documented. Univariable logistic regression models were used to assess associations between patient characteristics and 30-day mortality. Results are reported as odds ratios (ORs) with corresponding 95% confidence intervals (CIs) and p-values. Due to the limited sample size, multivariable analysis was not performed. Statistical significance was defined as a two-sided p-value < 0.05. Statistical analyses were performed using R version 4.3.2 (R Statistical Software Vienna, Austria).

## Results

**Figure 1:**
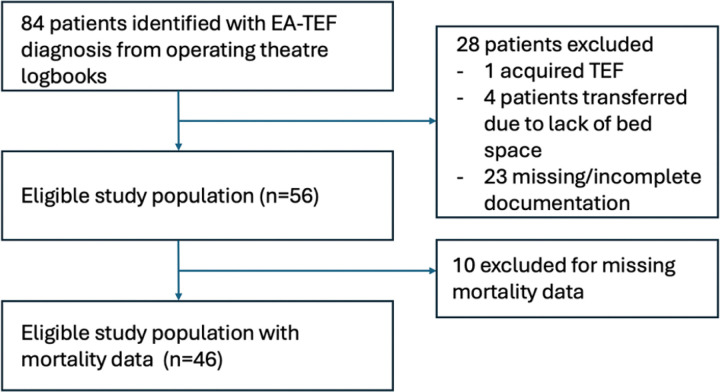
Flowchart demonstrating the definition of the study population and reasons for exclusion from our analysis.

Figure 1 demonstrates the patients included in our study. Eighty-four patients with a diagnosis of EA/TEF treated at CHUK from January 2015 to December 2023 were identified. Twenty-eight patients were excluded from our analysis: 1 had acquired tracheoesophageal fistula as the only diagnosis, 23 had inaccurate/inconsistent, or missing documentation, and four patients who were offered surgical repair at CHUK but were ultimately transferred to another hospital due to critical care bed availability. One patient had received a diverting colostomy prior to transfer to CHUK, but was not excluded as this was unrelated to EA/TEF repair. Our final study population was 56 patients, and all surgeries were performed by fellowship-trained pediatric surgeons.

Table 1 summarizes the demographic and clinical characteristics of the 56 patients. Any characteristics with missing information are also indicated. Among patients in our study population, 27 (48%) were male and 29 (52%) were female. Average birth weight was 2520g (638). Median gestational age was 40 weeks (36, 40). 36 (64%) patients were born in a health facility. Eight patients (14.3%) had missing information for place of birth. Fifty-one patients (96%) had health insurance. Fifty (98%) patients were born to mothers who had at least one prenatal visit during their pregnancy, while 32 (67%) had at least one prenatal scan. 43% of patients in our study resided in a region less than 50km from the tertiary facility. 21% lived greater than 50 kilometers but less than 100 kilometers away, and 36% lived more than 100 kilometers away.

The mean age upon arrival at CHUK was 7 days (SD 5.2), while the mean weight upon arrival was 2410g (SD 646). Type C EA/TEF was the most common type identified in this patient population, affecting 38 (68%) patients, followed by type A (isolated atresia) in 7 patients (12.5%), and type B in 1 patient (2%). The type of EA/TEF was missing from the medical records for 10 patients (18%). In our study cohort of 56 patients, 30 (54%) infants had an additional congenital anomaly of any type, and of that subset, 23 patients (41%) had a cardiac anomaly.

During the study period, infants evaluated at CHUK underwent various types of surgical interventions. Of the cohort, 49 patients (87.5%) underwent definitive repair of their EA/TEF. Eight patients (14.3%) had gastrostomy tube placement, either with or without a diverting esophagostomy. One patient (1.79%) underwent only a thoracotomy with repair of a gastric perforation. However, it is unclear whether that patient also had a laparotomy.

Mortality data were available for 46/56 (82.1%) patients. 30-day mortality was ascertained through either a filed death certificate, ongoing inpatient status, or recorded outpatient follow-up at CHUK that exceeded 30 days postoperatively. 24 patients (52%) died within 30 days of their initial surgical procedure

Table 2 summarizes the frequency of post-operative complications. The anastomotic leak rate was 29.2%. Esophageal stricture or stenosis affected 14.6% of patients. In comparison, recurrent fistula was observed in 4.8% of those who underwent complete repair and in 14.3% of patients who received fistula ligation without end-to-end anastomosis. Other postoperative complications, detailed in Table 2, included sepsis, which was the most common at 66%, and cardiopulmonary arrest, occurring in 30.4%. Among the 56 patients with EA, 3 (5.3%) experienced no complications—2 had EA/TEF repair for type C EA/TEF, and 1 had gastrostomy with esophagostomy for type A EA/TEF.

A summary of the univariate analysis of patient clinical and demographic characteristics’ effect on mortality can be found in Table 3. There was no significant association between mortality outcome and sex at birth, birth weight, distance travelled from the region of primary residence, age at surgery, type of EA/TEF, type of surgery performed, or time from admission to surgery. 75% of patients who died within 30 days of surgery had an additional congenital anomaly compared to 6% among those who were alive within 30 days of surgery (p=0.001). Similarly, there was a significant association between mortality and the presence of any congenital cardiac anomaly; 58% of patients who died within 30 days of surgery had an additional congenital anomaly compared to 18% among those who were alive beyond 30 days of surgery (p=0.01).

Furthermore, Table 3.1 summarizes the significant association between mortality and post-operative morbidity specific to EA/TEF repair, including anastomotic leak, recurrent fistula, and esophageal stricture or stenosis (p < 0.05), and other surgical morbidity, including sepsis, pneumonia, cardiopulmonary arrest, respiratory failure, and acute liver injury. Further analysis of predictors of mortality with univariate regression analysis is shown in Table 4. In a similar pattern, the following were all significant predictors of mortality (p<0.05): the presence of an additional congenital anomaly and more specifically the presence of a congenital cardiac anomaly, generally known complications after EA repair, and the presence of other post-operative complications specified in Table 2.

## Discussion

The clinical outcomes of patients with EA/TEF are frequently employed as an indicator of a nation's healthcare infrastructure and accessibility. (13) This study provides an analysis of the incidence, current surgical management, and post-operative outcomes of infants with esophageal atresia with /without tracheoesophageal fistula at CHUK, the largest tertiary referral and teaching hospital in Rwanda. The incidence of EA/TEF in Rwanda is not well documented in the literature. An epidemiological review of pediatric surgical care at two tertiary referral centers in Rwanda—CHUK and Butare University Teaching Hospital—over one year from 2013 to 2014 showed that 905 children received surgical care at CHUK and 369 children at Butare, with congenital anomalies accounting for nearly 25% of the surgeries performed (14). These findings demonstrate that congenital anomalies are frequently encountered in specialized hospitals in Rwanda and highlight the importance of pediatric surgeon expertise.

Overall, 30-day mortality in this study cohort was 52%. This mortality rate is higher than that observed in HICs, which have reported mortality rates for EA/TEF between 5% and 15%(6)(8)(15)(16). However, this finding reflects more favorable outcomes of EA/TEF in Rwanda compared to other LMICs.(17)(18)(19)(20). A study in Bangladesh reported an EA/TEF mortality rate of 88%. Another study in Senegal found it to be 71%. A study involving 225 neonates in Ethiopia documented a rate of 71%, and a different study reported 74% in Benin(21)(22)(23). Type C EA/TEF was the most common type observed in this population, affecting 68% of patients, which is lower than the expected prevalence of type C reported in the literature at 84 – 86%(2)(24)(25)(26). This finding may be attributable to incomplete information in patients’ medical records, considering that this variable was absent in the records of ten patients. The presence of an additional congenital anomaly, and more specifically the presence of a cardiac congenital anomaly, was the only predictor in this study. Similar results have been reported in other studies demonstrating a similar association between other congenital anomalies and mortality in EA/TEF(3)(27)(28). Historically, corrective surgery for congenital cardiovascular anomalies in Rwanda relied on visiting surgeons who were in the country for short periods of time to perform surgeries. Still, since 2022, a dedicated cardiac surgery program has been established with a full-time pediatric cardiac surgeon, treating a wide range of congenital heart defects since its inception(29). Partnerships with congenital cardiac specialists and further studies on the types of cardiac anomalies with the highest risk are needed to reduce the associated increased risk potentially.

Sepsis was a leading cause of morbidity in this cohort, affecting 69.4% of patients (n=34/49) who underwent a definitive repair or trachea-esophageal fistula closure. An increased mortality due to sepsis has been shown in the literature in LMICs, and in a large international study of gastrointestinal congenital anomalies in 74 countries, Wright et al reported a higher proportion of patients presenting with sepsis in LMICs compared to HICs. While sepsis was not associated with mortality in HICs, it was significantly associated with mortality in LMICs(8). Additionally, a retrospective review of infants with gastroschisis and intestinal atresia in Rwanda by Davis et al. reported that sepsis was present in 100% of patients who died within 48 hours of presentation compared to 46% who survived beyond 48 hours(30). The association between sepsis and mortality in this population has been previously shown(28)(31). The potential need for mechanical ventilation, central venous catheters, and the potential need for invasive devices in these postsurgical patients can also be predictive factors for infections and increase the risk of sepsis (32). Further studies are needed to identify the leading causes of sepsis in this population and to develop standards for sepsis management that can improve outcomes for infants with EA/TEF, supporting the development of targeted interventions.

This study has several potential limitations. First, it is retrospective in nature and subject to inherent bias, including selection and information bias. We identified over 80 patients; however, a significant number were excluded due to missing or incomplete information. This study was also conducted in a single center, resulting in a small sample size. Thus, it is more challenging to observe true relationships between the outcome of interest and predictive variables. Additionally, data was collected from paper medical records, which were all handwritten. Legibility was sometimes challenging, and there were frequent occurrences of missing or incomplete documentation and differences in documentation style among providers.

## Conclusion

This study highlights the improved mortality outcomes, driven mainly by sepsis and ventilator-associated complications, in EA/TEF management due to significant changes that occurred in the health system capacity, such as the availability of a fellowship-trained pediatric surgeon, a dedicated pediatric operating room, and expansion of neonatal and pediatric intensive care units in a low-income country. This represents an improvement based on what has been reported in LMICs. Targeted strategies for infants with EA/TEF and other associated congenital anomalies, especially cardiac anomalies, could further improve outcomes. Additional strategies to improve outcomes include developing standardized bundles for sepsis management within the limitations of available resources.

## Figures and Tables

**Table 1:** Infant Demographic and Clinical Characteristics

Birth weight in grams, mean(SD)↷	2520 (638)
Gestational age in weeks, median (IQR)	40 (36, 40)
Age upon arrival to tertiary hospital in days, median(IQR) ^	5 (4,10)
Weight upon arrival to tertiary hospital in grams, mean(SD)⊠	2410 (646)
Sex, n (%)	
Male	27 (48)
Female	29 (52)
Distance from region of primary residence to CHUK, n (%)	
< 50km	24 (43)
50km - 100km	12 (21)
>100km	20 (36)
Prenatal visit (at least 1), n (%) ↬	
Yes	50 (98)
No	1 (2)
Prenatal scan (at least 1), n (%) ⊠	
Yes	32 (67)
No	16 (33)
Insured, n (%) ⊠	51 (96)
Birth location, n (%) ⊠	
Health care facility	36 (64)
Other	12 (21)
Type of EA/TEF, n (%) ↟	
Type A	7 (12)
Type B	1 (2)
Type C	38 (68)
Additional congenital anomaly, n (%)	30 (54)
Cardiac congenital anomaly, n (%)	23 (41%)
Death within 30 days of initial surgery, n (%) *	
Yes	24 (52)
No	22 (48)

* mortality data available for only 46 patients.

↷ 4 missing

^ 2 missing

⊠ 3 missing

↬ missing

⊠ 8 missing

↟ 10 missing

⊠ 17 missing

**Table 2:** Post-operative outcomes by type of surgery offered

	Primary repair n = 42 (%)	Fistula ligation without anastomosis* n = 7 (%)	Gastrostomy tube only, n= 7 (%)
Repair-related postoperative complications			
Anastomotic leak	12 (29.2%)	•	•
Stricture/stenosis	6 (14.3%)	•	•
Recurrent fistula	2 (4.8%)	1 (14.3%)	•
Other postoperative complications			
Sepsis	28 (66.7%)	6 (85.7%)	3 (42.9%)
Pneumonia	11 (26.2%)	1 (14.3%)	1 (14.3%)
Renal injury	4 (16.7%)	1 (14.3%)	0 (0.0%)
Respiratory failure	10 (23.8%)	2 (28.6%)	1 (14.3%)
Cardiopulmonary arrest	11 (26.2%)	3 (42.9%)	3 (42.9%)

* includes children who also had a combined gastrostomy tube or diversion esophagostomy without an anastomosis

**Table 3:** Univariable analysis of patient characteristics impact on 30-day mortality

30-day mortality				
Variable		Yes (n=24)	No (n=22)	p-value
Sex, n (%)	Male	11 (45.8)	13 (59.1)	0.37
	Female	13 (54.2)	9 (40.9)	
Birth weight in grams,, n (%)				0.681
	<2000	8 (34.8)	4 (21.1)	
	2000 – 2999	8 (34.8)	8 (42.1)	
	>3000	7 (30.4)	7 (36.8)	
Distance travelled in km,, n (%)	<50	11 (45.8)	8 (36.4)	0.36
	50–100	7 (29.2)	4 (18.2)	
	>100	6 (25.0)	10 (45.5)	
Age at initial surgery in days, n (%)				
	<7	9 (37.5)	4 (18.2)	0.39
	8–14	11 (45.8)	13 (59.1)	
	>14	4 (16.7)	5 (22.7)	
Health Insurance, n (%)				
	Yes	23 (100)	18 (90)	0.21
	No	0 (0)	2 (10)	
Type of EA/TEF, n (%)				
	Type C	17 (70.8)	14 (63.6)	0.84
	Other (A or B)	3 (12.5)	4 (18.2)	
	Not reported	4 (16.7)	4 (18.2)	
Type of surgical intervention offered at CHUK, n (%)				
	Primary repair	18 (75.0)	14 (63.6)	0.4
	Other surgery	6 (25.0)	8 (36.4)	-
Time from admission to surgical intervention in days, n (%)				0.6
	<2	12 (50)	8 (36.4)	
	3–6	8 (33.3)	8 (36.4)	
	>6	4 (16.7)	6 (27.2)	
Presence of any congenital anomaly, n (%)				
	Yes	18 (75.0)	6 (27.3)	**0.001**
	No	6 (25.0)	16 (72.7)	
Presence of cardiac congenital anomaly, n (%)				**0.014**
	Yes	14 (58.3)	4 (18.2)	
	No	10 (41.7)	18 (81.8)	

* All percentages are of those reported. Missing data is denoted in Table 1.

**Table 3.1:** Surgical morbidities associated with 30-day mortality after EA/TEF repair

		30-day mortality		

Variable		Yes	No	p-value

Presence of EA/TEF-repair associated morbidity, n (%)↮	Yes	3 (14.3)	13 (72.2)	**0.0003**
	
	No	18 (85.7)	5 (27.8)	

Presence of other post-operative morbidity, n (%)⊠	Yes	20 (95.2)	10(55.6)	**0.0006**
		
	No	1(4.8)	8(44.4)	
		

↮ includes anastomotic leak, anastomotic stricture or stenosis, and recurrent fistula.

⊠ post-operative morbidity includes sepsis, pneumonia, cardiopulmonary arrest, respiratory failure, and renal injury.

**Table 4:** Univariable Logistic regression for predictors of mortality.

Variable	n = 46	OR (95% CI)	p-value
Sex, n (%)			
Female	22 (47.8)	1.71 (0.53, 5.50)	0.37
Male	24 (52.2)	1.00	
Birth weight in grams ↷			
<2000	12 (28.6)	2.00 (0.41, 9.84)	0.39
2000–2999	16 (38.1)	1.00 (0.24, 4.20)	>0.999
>=3000	14 (33.3)	1.00	
Gestational age <37 weeks⊠	10 (30.3)	2.55 (0.52, 12.37)	0.25
Distance traveled in kilometers			
<50	19 (41.3)	2.92 (0.59, 8.94)	0.23
50 – 99	11 (23.9)	2.92 (0.59, 14.33)	0.19
>=100	16 (34.8)	1.00	
Type of EA/TEF			
Type C	31 (81.6)	1.00	
Non-Type C	7 (18.4)	0.61 (0.11, 3.26)	0.57
Weight at surgery (g)			
<2000	10 (30.3)	1.50 (0.26, 8.82)	>0.999
2000–2999	13 (39.4)	1.17 (0.22, 6.08)	
>=3000	10 (30.3)	1.00	
Additional congenital anomaly	24(52.2)	8.00 (2.14, 29.85)	**0.002**
Presence of cardiac anomaly	19 (41.3)	4.76 (1.32, 17.22)	**0.017**
EA/TEF-repair associated morbidity	39 (84.8)	0.06 (0.01, 0.28)	**0.0007**
Other post-operative morbidity	39 (84.8)	16 (2.45, 318.97)	0.014

↷ 4 missing

⊠ 13 missing

## Data Availability

De-identified datasets used and analyzed in this study are available from the corresponding author upon reasonable request.
